# Does health insurance coverage reduce informal payments? Evidence from the “red envelopes” in China

**DOI:** 10.1186/s12913-020-4955-7

**Published:** 2020-02-06

**Authors:** Ning Liu, Guoxian Bao, Alex Jingwei He

**Affiliations:** 1 School of Management and Research Center for Hospital Management, Lanzhou University, 222 Tianshui South Road, Lanzhou City, Gansu Province China; 2 Department of Asian and Policy Studies, The Education University of Hong Kong, 10 Lo Ping Road, New Territories Tai Po, Hong Kong

**Keywords:** Informal payments, Health insurance, Corruption, China, Health policy

## Abstract

**Background:**

Representing a major threat to both equity and efficiency of health systems, the corrupt practice of informal payments is widely found in developing and transition countries. As informal payments are more likely to occur in health systems characterized by a high out-of-pocket payment rate, it is argued that formalized prepaid health insurance programs may help to curb such practice.

**Methods:**

Using panel data from the China Health and Retirement Longitudinal Survey, this study examined the association between changes in health insurance coverage on patient’s behavior proxied with informal payments.

**Results:**

The statistical results reveal that health insurance status in fact increases the probability of patients making informal payments to physicians. However, this association varies among population groups and insurance programs, particularly between social health insurance and private health insurance status*.*

**Conclusions:**

In a health system characterized by unequal allocation of medical resources, the dual pursuit of cost saving and quality of care may drive patients to make informal payments for personal gains. This study argues that health policy interventions aimed at curbing informal payments must be based on a thorough understanding of their complex socioeconomic causes and attack the perverse incentives in a coherent and bona fide manner.

## Background

Informal payments from patients to physicians are prevalent in the health systems of developing and transition countries. Informal payments are defined as payments to individuals or institutions, in cash or in kind, made outside official payment channels for services that are meant to be covered by the health system [1]. It was estimated that informal expenditures formed a sizable proportion of healthcare financing in former Eastern European communist countries [2, 3]. Typically involving additional payments to healthcare providers for services that patients are entitled to, informal payments are commonly regarded as an illegal corrupt practice [4]. Various studies have suggested that informal payments increase the financial burden of patients, distort the behavior of physicians [5, 6] and undermine the efficiency and equity of health system [7, 8]. The perverse incentives nurtured along the line of such transactions may forge strong vested interests on the part of medical professionals that ultimately thwart necessary healthcare reforms [9].

A large body of literature has sought to explain the mechanisms underlying informal payments. Major theoretical frameworks include cultural tradition [2], institutional deficiency [7], doctor-patient relationship and trust deficit [10, 11], weak professional ethics of physicians [6], and so forth. Because informal payments are more likely to occur in health system characterized by a high out-of-pocket payment rate, it is argued that formalized financing and payment mechanisms, such as prepaid health insurance programs, may help curb or eliminate private payments [10, 12, 13]. The empirical evidence received thus far, however, suggests that the increase of insurance coverage and the arrangement of coinsurance have had a limited effect in containing informal payments [5, 14, 15].

This present study joins the scholarly debate with empirical evidence from China, which provides a unique setting to examine the relationship between health insurance and informal payments in a developing country. Commonly referred to as “red envelopes” (*hongbao*), informal payments are prevalent in the Chinese health system [11]. Despite their cultural connotation as an expression of gratitude, red envelopes hardly existed in China during the communist planned economy period when most residents were poor but were covered by basic health insurance schemes. Following the structural changes in the health system in 1980s and 1990s, most residents were left uninsured while access to health services deteriorated. Red envelopes paid to physicians resurged quickly in the Chinese society [10].

The past two decades have witnessed the rapid expansion of social health insurance (SHI) in China, thanks to the central government’s strong political will and fiscal strength [16, 17]. Two major SHI schemes have together covered almost the entire population, despite certain drawbacks such as systemic fragmentation and entitlement disparities among different groups of the population [17, 18]. Against the backdrop outlined above, namely the increasing prevalence of informal payments occurring largely hand in hand with the expansion of SHI, China offers an ideal dynamic setting to investigate whether growing SHI coverage has led to discernable changes in the prevalence of informal payments.

To fulfill this research mission, we draw on three-phase panel data (2011–2015) from the China Health and Retirement Longitudinal Survey (CHARLS) to analyze whether health insurance coverage and its dynamic changes have increased or decreased the chance of Chinese patients making red envelope payments to physicians. The results suggest that being insured, especially by SHI, increases the probability of patients making informal payments. Yet, the power of this effect varies between social insurance and private insurance status and between actual SHI schemes. Using the concept of budget constraint, we interpret this variation as resulting from the varying degrees of budget constraint confronted by patients, which may, in turn, alter their cost-benefit calculation.

This study contributes to the literature by shedding fresh light on informal payments by illustrating the role played by health insurance in a developing transition economy. The remainder of this article proceeds as follows. Section 2 describes the context of the research, that is, the Chinese health system and the phenomenon of red envelopes. Research hypotheses are formulated in this section too. The research methodology and empirical results are reported in Section 3 and Section 4, respectively, followed by robustness checks of the results in Section 5. The last section concludes the article with a discussion on policy implications.

## Research context and hypotheses

### Historical background

China used to have a well-functioning health system prior to embarking on market-oriented reforms in 1980s. The urban-rural dichotomy was reflected in the institutional arrangement of health system [19]. Two government-organized health insurance programs, namely, the Government Insurance Scheme (GIS) and the Labor Insurance Scheme (LIS) covered majority of the urban population, while the Cooperative Medical Scheme (CMS)—a community risk-pooling program—insured the rural population [20]. The service delivery system was entirely public between 1949 and 1980s. In the urban health system, services were provided through a three-tier network consisting of public hospitals and community health stations. Township health centers and barefoot doctors formed the cornerstone of the rural system [19]. Under the communist planned economy, planning and resource allocation of the health system were subject to tight central command-and-control. The entire sector was taken as part of the broader quasi-government system [10, 21]. Health professionals in the cities were state employees, receiving a fixed but decent income, while the rural barefoot doctors were paid through the CMS [22]. With private medical practice outlawed since 1949, virtually no market mechanisms operated in China’s health system until 1980s [21]. The communist medical training upheld—at least in principle—superior professional morality that was by no means compatible with informal payments. In short, neither the communist ideology nor the financial incentives allowed informal payments [7], although offering red envelopes to physicians had a long history in China [10].

This communist health financing regime was swiftly dismantled in the 1980s following the structural changes in the economy. The market-oriented health reforms embraced market rules that replaced central planning. The rapid collapse of CMS in rural China in the early 1980s and the mass lay-off of state-owned enterprise employees in the 1990s led to the deprivation of financial protection against catastrophic diseases for hundreds of millions of people [19, 23]. By 1998, the proportion of the population covered by health insurance dropped to 44.1% in urban areas [23] and to a mere 4.7% in rural areas [24].

In the meantime, drastic changes in the provision system further reduced financial accessibility to numerous patients. Marketization encouraged hospitals and physicians to pursue profits while the nominal salary of health professionals was kept low. While the underfunded public hospitals started to pursue revenues, the entire incentive structure in the Chinese health system heavily skewed towards profit making [25]. The outcomes were the well-known double-digit escalation of healthcare costs, the vast provision of unnecessary care, and the resultant medical impoverishment for numerous households [22, 26].

### Informal payments in China

This unregulated marketization on the supply-side and the loss of financial protection on the demand side together fueled competition among patients for high-quality medical services. As a result, red envelopes had gradually become a popular informal mechanism to coordinate the supply and demand of scarce medical resources between physicians and patients [21]. Informal payments in China predominantly take the form of red envelope payments to physicians in cash. It has been estimated by several nationwide surveys that the rate of red envelope payments varies between 54.4% [27] and 76.1% [28]. In the Chinese society, red envelopes were sometimes meant to express gratitude under certain circumstances [11], but the prevalence varied across gender, age, occupation, and regions [27, 28]. Since the 1990s, the Chinese government has explicitly defined the illegality of red envelops as corruption, and therefore, their connotation as a presentation of gratitude to physicians has been diminishing [29].

In short, two key factors on both the supply side and the demand side explain the prevalence of red envelopes in China despite their illegality. First, the metropolis-skewed allocation of medical resources essentially leads to a scarcity of such resources for numerous patients in rural areas and small cities. Bribes have thus become an effective way to secure access to services provided by physicians with higher qualifications. Offering red envelopes was further encouraged by people’s rising ability-to-pay and their expectations for quality care. Second, the average nominal salary of Chinese medical professionals has been remarkably low, so they earn additional income from various sources, such as bonuses, drug commissions, and bribes [30, 31]. The decline of medical ethical standards in the past three decades relaxed the self-discipline of millions of underpaid Chinese physicians with regard to accepting red envelopes, while both bona fide legal enforcement and professional self-regulation are significantly weak [22, 30].

### The social health insurance system

**Fig. 1:**
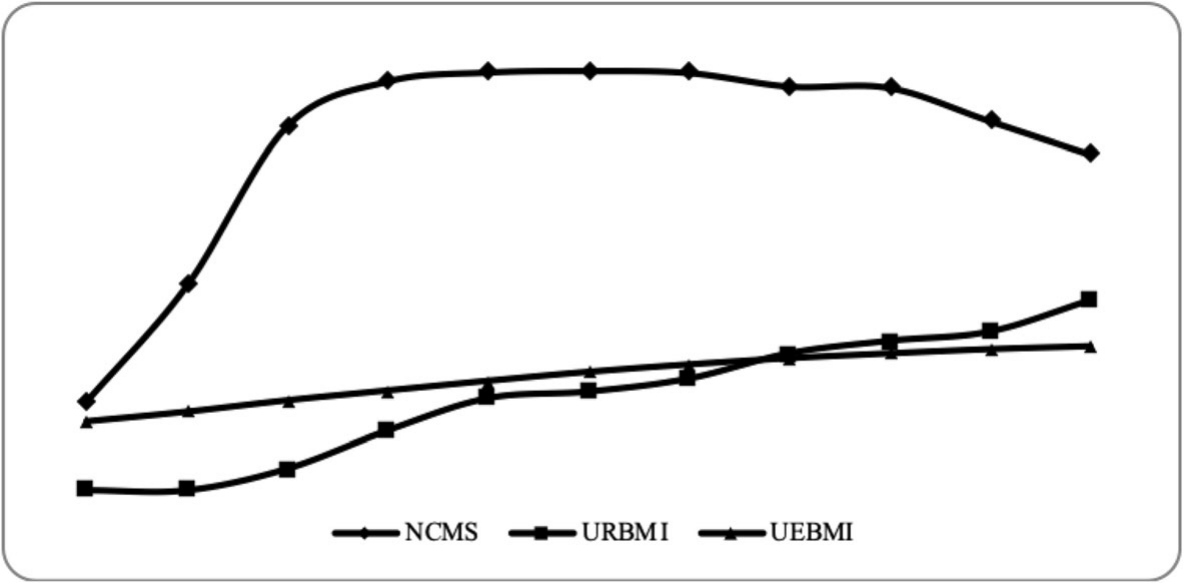
Coverage of social health insurance programs in China, 2005–2015 (Unit: 100 million). Source: China Health Statistical Yearbook 2016

SHI is the most salient feature of China’s health financing system. Two schemes insure urban employees and the rest of the population, respectively. The Urban Employee Basic Medical Insurance Scheme (UEBMI) is financed by contributions from both employers and employees on a fixed formula. The Urban-Rural Resident Basic Medical Insurance Scheme (URRBMI) was merged from the former New Cooperative Medical Scheme (NCMS) and the Urban Resident Basic Medical Insurance Scheme (URBMI). Financed by personal contributions and generous government subsidies, this scheme covers the vast rural population and urban residents outside the formal sector. The two schemes together have helped China to make impressive strides towards universal insurance coverage [29] (See Figure 1 below). It must be pointed out that the SHI system in China is organized in a decentralized fashion. Most schemes are operated at the prefectural level, creating a rather fragmented system. There are large disparities in terms of financing formula and benefit package among local SHI schemes [29]. Mainly catering to the affluent segments of society, private health insurance plays a supplementary role in China’s health financing [29].

### Hypotheses

The role of health insurance on individual health status, household income, and national wealth have been well documented in the literature [32–34]. It is generally agreed that health insurance alleviates—at least partially—the budget constraints of individuals [35]. The empirical evidence gleaned thus far suggests that health insurance helps to reduce poverty [36], increase household savings [37], and encourage long-term household investment, such as on education and children’s health [38, 39]. By the same token, the relaxed budget constraints because of insurance coverage may lead patients to make informal payments in return for a variety of personal gains, such as faster access to care [2] and higher quality (at least perceived) care [12], among other benefits. Consequently, we propose the following hypothesis:

*H1: When covered by health insurance, which may relieve one’s budget constraints, a patient is more likely to make informal payments for personal gains.*

The type of health insurance may alter the probability of a person making informal payments. These hierarchical effects stem from the differences among health insurance programs in terms of coverage and copayments [13, 40]. In China, SHI covers a significant portion of the entire population whereas private health insurance (PHI) plays a supplementary role. Typically associated with low participation, high premiums, and a high reimbursement standard, PHI serves the few residents who are ineligible for any SHI but are usually poor and vulnerable, as well as middle- and high-income residents who hold SHI but seek additional risk protection [41–43]. Against this backdrop, the variety of health insurance schemes has essentially divided the Chinese population into four groups: 1) the group covered by SHI, 2) the group with PHI, 3) the group with both SHI and PHI, and 4) the group without any health insurance. We thus propose the following hypothesis:

*H2: The effects of health insurance on informal payments by patients vary among population groups and insurance programs, particularly between SHI and PHI.*

The group covered by SHI only constitutes the majority of the population in China who typically seek a trade-off between low cost but high quality care [36, 44, 45]. Evidence from other health systems primarily financed through SHI suggests that informal payments may be encouraged by the financial payouts aided by SHI [13, 14, 46]. The situation is quite similar in China: Many expensive medical procedures and pharmaceuticals are excluded from SHI reimbursement but may be unnecessarily prescribed to patients, who therefore have the incentive to either offer informal payments in order to avoid over-prescription or seek alternative arrangements. When the financial protection provided by the SHI program is weak, a patient may have an even stronger incentive to make informal payments. We hence posit the following hypothesis for empirical testing:

H2a: *SHI increases the probability of a patient making informal payments, and the effect may be amplified by less generous insurance programs,* i.e.*, those that involve high out-of-pocket payments.*

Purchasers of PHI in China represent two different groups of individuals: those insured by SHI and those without any SHI. The latter are usually ineligible for any SHI but may subscribe to a certain high-cost PHI program in order to reduce catastrophic risks [47]. Mainly comprised of the migrant population, this group also typically pursues the trade-off between low cost but high quality care [43, 48]. PHI has been found to be able to curb informal payments because of its higher reimbursement rate and wider coverage of expensive procedures and pharmaceuticals [13, 43, 48]. Hence, we propose the following hypothesis:

*H2b: PHI decreases the probability of a patient making informal payments among the population covered by PHI only.*

When insured by both SHI and PHI, a patient’s budget constraint is further relaxed and he/she may be more likely to seek higher quality care and greater convenience, making informal payments an appealing option. The same phenomenon is observed in some Western European health systems where patients pay informally to skip the waiting list in order to access high-quality care or advanced medical technologies and medicines [49]. Therefore, we pose another hypothesis in the Chinese context for empirical testing:

*H2c: The dual coverage of SHI and PHI increases the probability of a patient making informal payments, and this effect is greater than that for a patient with SHI coverage only.*

## Methods

### Data

The individual-level panel data used in this study came from the CHARLS database, a nationally representative longitudinal survey of Chinese citizens aged 45 or older and their spouses. Including assessment of socioeconomic and health circumstances of respondents, this survey examines individuals’ health and economic adjustments to rapid ageing population [50]. This nationwide survey covers 28 provinces in mainland China. In this present study, we extracted data from three waves, 2011, 2013, and 2015, yielding a total sample of 76,512 respondents that included a sample of 7069 with inpatient experience.

### Variables

The dependent variable was measured by whether or not the respondent had made red envelope payments to a medical doctor at his/her most recent inpatient visit in the past 12 months. Although self-reported measurement is prone to underestimating the real incidence of informal payments, the reliability of the CHARLS data has been widely recognized [51–53]; thus far, CHARLS is the only national database that contains information about informal payments in China.

The explanatory variables related to health insurance status included: 1) whether the respondent was covered by any health insurance program, 2) whether he/she was covered by SHI or by PHI, and 3) whether he/she was insured by UEBMI or NCMS. Then, we used the interaction between coverage of any health insurance program and fixed year as another explanatory variable to capture the potential time-dynamic variation of effects. The control variables were social and demographic characteristics of patients, such as age, gender, occupation, and marital status. We also controlled the region of residence (urban or rural), and health status of patient by the Activities of Daily Living (ADL) indicators. These covariates were used to capture the potential contaminations from residential and personal characteristics [27, 28]. Variable definitions are set out in Appendix Table 8.

### Models

With the aforementioned dataset that covers 28 provinces in China, we were able to measure a patient’s self-reported red envelope payments for hospital care. We first distinguished a person’s health insurance status and the actual scheme to which he/she subscribed in order to provide a more nuanced analysis of the impact of SHI on a patient’s behavior of informal payments. In statistical analysis, two estimation strategies were employed. First, the effect of the dynamic change in health insurance coverage over time was estimated. Second, the linear probability and random effects models were used to estimate the static effect of health insurance coverage.

We began by estimating the dynamic effect of health insurance status on a person’s decision to make informal payments. We compared changes in the probability of a patient making red envelope payments over time, with the year 2011 as the baseline. Our estimation equation is as follows:

1 $$ {Y}_{ijt}=\boldsymbol{\upalpha} +{\boldsymbol{\upgamma}}_j+{\boldsymbol{\uplambda}}_t+{\boldsymbol{\updelta}}_{jt}+{\mathbf{X}}_{ijt}{\beta}_1+{\beta}_2 in{s}_{ijt}+\mathbf{tin}{\mathbf{s}}_{ijt}{\beta}_3+{\boldsymbol{\upvarepsilon}}_{ijt}, $$

where *i* represents the individual patient, *j* denotes the province, and *t* represents the year. The variable *Y*_*ijt*_ denotes the binary outcome of a red envelope payment for patient *i* from province *j* in year *t*. The binary viable *ins*_*ijt*_ denotes the coverage of any health insurance. The vector **X**_*ijt*_ represents a set of individual-level covariates that may affect the behavior of making red envelope payments.

The target variable **tins**_*ijt*_ is the interaction term of *ins*_*ipt*_ with year *t*, and it is a vector containing two variables because of the three-phase structure of our dataset. The coefficient of interest is represented by *β*_3_, reflecting the extent to which the dynamic change in coverage for any health insurance affects the patient’s behavior of making red envelope payments. **γ**_*j*_ refers to regional fixed effects at the provincial level to control for factors such as poverty and cultural differences that do not change significantly over time. **λ**_*t*_ denotes the time-fixed effect that is included to control for the unobserved effects of individual characteristics over time. **α** and **ε**_*ijt*_ are the constant term and the error term, respectively. The variable **δ**_*jt*_ represents the regional-by-year fixed effects.

In all the statistical analyses, the covariates included the demographic characteristics of patients, such as age and gender, total household assets, and annual household income. A patient’s health status was controlled by using the Activities of Daily Living (ADL) indicators. Representing a person’s official residential status, *hukou* (household registration status) was controlled. A person’s place of permanent residence (rural or urban) was also included to control for possible urban-rural differences in outcomes. We further controlled a person’s history of inpatient care, as well as the characteristics (public or private and level) of the health facilities visited most frequently by the respondent.

There are significant differences between SHI and PHI programs in China in terms of financing formula, administration, and reimbursement rate. Therefore, we used model (2) and model (3) to identify the static impact of health insurance coverage on the behavior of making red envelope payments in order to distinguish the potential differences (a) between these two types of health insurance and (b) among different SHI schemes.

2 $$ {Y}_{ijt}=\boldsymbol{\upalpha} +{\boldsymbol{\upgamma}}_j+{\boldsymbol{\uplambda}}_t+{\boldsymbol{\updelta}}_{jt}+{\mathbf{X}}_{ijt}{\beta}_1+{\beta}_2 go{v}_{ijt}+{\beta}_3 pri{v}_{ijt}+{\boldsymbol{\upvarepsilon}}_{ijt}, $$

3 $$ {Y}_{ijt}=\boldsymbol{\upalpha} +{\boldsymbol{\upgamma}}_j+{\boldsymbol{\uplambda}}_t+{\boldsymbol{\updelta}}_{jt}+{\mathbf{X}}_{ijt}{\beta}_1+{\beta}_2 go{v}_{ijt}+{\beta}_3 pri{v}_{ijt}+{\beta}_4 go{v}_{ijt}\times pri{v}_{ijt}+{\boldsymbol{\upvarepsilon}}_{ijt}. $$

In model (2) and model (3), *gov*_*ijt*_ and *priv*_*ijt*_ represent the coverage of SHI and PHI, respectively. *gov*_*ijt*_ × *priv*_*ijt*_ is the interaction term of *gov*_*ijt*_ and *priv*_*ijt*_. The other variables are the same as in model (1). The coefficients of interest are represented by *β*_2_, *β*_3_, and *β*_4_, which identify the extent to which the coverage of SHI, PHI, and both SHI and PHI, respectively, changes a patient’s behavior of red envelope payments.

We further selected the UEBMI and NCMS through models (2) and (3) to investigate the possible disparities between the urban and rural healthcare markets in China and between different SHI schemes. To control for potential estimation biases, we first performed a linear probability model (LPM) estimation with clustering within households in each model. Secondly, we considered the fixed effect and random effect models as the strategies to correct the estimation bias by using panel data. However, we were constrained by the problem of high consumption for degree of freedom; no convergence was noted when estimating the fixed model for some variables. In the fixed models that converged, the Hausman test revealed that the random effect model was more robust in this estimation.

## Results

A descriptive summary of the key variables is presented in Table 1. In the sample, 7069 respondents reported at least one inpatient admission in the past 12 months, while the incidence of self-reported informal payments was 1.8%, on average, among all respondents. SHI and PHI covered 82.7 and 2.4% of the respondents in the sample, respectively, and 92.5% of the inpatient stays occurred in public hospitals.

**Table 1:** Descriptive results of key variables

Variables	#total sample			#inpatient sample		
	N	Mean	S. D	N	Mean	S. D
SHI	76,512	0.927	0.260	7069	0.947	0.224
Year 2011	25,504	0.921	0.270	1654	0.948	0.221
Year 2013	25,504	0.953	0.150	2516	0.973	0.162
Year 2015	25,504	0.905	0.292	2899	0.920	0.271
PHI	76,512	0.024	0.152	7069	0.021	0.144
Year 2011	25,504	0.025	0.157	1654	0.026	0.160
Year 2013	25,504	0.023	0.150	2516	0.018	0.132
Year 2015	25,504	0.023	0.150	2899	0.022	0.147
UEBMI	76,512	0.943	0.232	7069	0.949	0.219
Year 2011	25,504	1.000	0.000	1654	1.000	0.000
Year 2013	25,504	1.000	0.000	2516	1.000	0.000
Year 2015	25,504	0.854	0.353	2899	0.881	0.324
NCMS	76,512	0.994	0.079	7069	0.995	0.074
Year 2011	25,504	1.000	0.000	1654	1.000	0.000
Year 2013	25,504	1.000	0.000	2516	1.000	0.000
Year 2015	25,504	0.981	0.137	2899	0.985	0.121
Red envelopes	76,512	0.018	0.134	7069	0.018	0.134
Year 2011	25,504	0.022	0.147	1654	0.022	0.147
Year 2013	25,504	0.023	0.149	2516	0.023	0.149
Year 2015	25,504	0.012	0.110	2899	0.012	0.110

Table 2 reports the estimates of eq. (1). The results reveal a significant positive dynamic relationship between health insurance and a patient’s behavior of making red envelope payments over time. Specifically, for an uninsured patient, the probability of making informal payments after gaining insurance protection increased by 3.6% (2013) and 3.9% (2015). The probability of making red envelope payments after gaining SHI coverage increased by 3.5% (2013) and 3.8% (2015). Hypothesis 1 is hence supported.

**Table 2:** Statistical results: the time variation of health insurance and red envelopes

	Red envelopes
Panel A	
tins_2013_	3.615^***^
	(0.496)
tins_2015_	3.935^***^
	(0.515)
ins	0.0470
	(0.440)
N	1642
Panel B	
tins_2013_	3.494^***^
	(0.467)
tins_2015_	3.796^***^
	(0.489)
SHI	0.175
	(0.418)
N	1647
Panel C	
tins_2013_	–
	–
tins_2015_	–
	–
PHI	0.197
	(0.417)
N	1229
Province	Yes
Time	Yes
Prov×Time	Yes
Control	Yes

Note: * = *p* < 0.1, ** = *p* < 0.05, *** = *p* < 0.01; robust standard errors in parentheses

Derived from the estimation on eqs. (2) and (3), Table 3 suggests that SHI coverage significantly increases the probability of a patient making red envelope payments, while PHI coverage significantly reduces this probability. We also examined the behavior of patients insured by both SHI and PHI through the interaction term. It is noted that the probability of informal payments being made is significantly increased for this group of respondents, and the increase is greater than that for the group insured by SHI only.

**Table 3:** Statistical results: coverage of SHI and PHI, and red envelopes

	(1)	(2)	(3)	(4)	(5)	(6)	(7)	(8)
SHI	0.041	0.041^**^			0.032	0.032^**^	0.005	0.005
	(0.027)	(0.016)			(0.024)	(0.016)	(0.018)	(0.016)
PHI			−0.068^*^	−0.068^***^	− 0.061^*^	− 0.061^***^	− 0.254^*^	− 0.254^***^
			(0.038)	(0.021)	(0.033)	(0.021)	(0.140)	(0.038)
SHI× PHI							0.243^*^	0.243^***^
							(0.140)	(0.044)
N	1882	1882	1877	1877	1877	1877	1877	1877
Province	Yes	Yes	Yes	Yes	Yes	Yes	Yes	Yes
Time	Yes	Yes	Yes	Yes	Yes	Yes	Yes	Yes
Prov×Time	Yes	Yes	Yes	Yes	Yes	Yes	Yes	Yes
Control	Yes	Yes	Yes	Yes	Yes	Yes	Yes	Yes

Note: * = *p* < 0.1, ** = *p* < 0.05, *** = *p* < 0.01; robust standard errors in parentheses

We selected the NCMS and UEMI to investigate the nuanced effect of SHI coverage on informal payments by estimating eqs. (2) and (3). The benefit package of UEBMI is more generous than that of NCMS: for example, the former offers a higher rate and ceiling of reimbursement compared to the latter [29]. As shown in Table 4, the UEBMI group demonstrated a significantly lower probability of making red envelope payments, all other factors being equal. A notable finding is the positive effect of NCMS on red envelope payments, although the coefficient is not statistically significant; this probability was substantially reduced to negative if this group was also covered by PHI. Therefore, all of our hypotheses are supported.

**Table 4:** Statistical results: coverage of UEBMI, NCMS, PHI, and red envelopes

	UEBMI			NCMS		
	(1)	(2)	(3)	(4)	(5)	(6)
UEBMI	−0.039^*^	−0.040^*^	−0.040^*^			
	(0.030)	(0.032)	(0.032)			
NCMS				0.013	0.01	0.024
				(0.011)	(0.013)	(0.016)
PHI		−0.024	−0.024		−0.011^*^	0.030
		(0.017)	(0.017)		(0.006)	(0.019)
UEBMI×PHI			0.000			
			(0.000)			
NCMS×PHI						−0.042^**^
						(0.021)
N	1061	991	991	1848	1844	1687
Province	Yes	Yes	Yes	Yes	Yes	Yes
Time	Yes	Yes	Yes	Yes	Yes	Yes
Prov×Time	Yes	Yes	Yes	Yes	Yes	Yes
Control	No	No	No	Yes	Yes	Yes

Note: * = *p* < 0.1, ** = *p* < 0.05, *** = *p* < 0.01; robust standard errors in parentheses

## Robustness checks

Our statistical results might be biased by potential biases, for example, upwardly by unobservable factors correlated with the coverage of health insurances and with subsequent decision to deliver informal payments. Moreover, the payment of red envelops during catastrophic diseases may be a household decision rather than an individual decision. Thus, the potential selection bias may also affect our results.

In order to corroborate our statistical results, we pursued four strategies to assess whether the correlations documented to this point were robust. First, we used selection on observable variables to assess the likelihood that our estimates were driven by unobserved heterogeneity. Second, we constructed a household dataset to assess potential bias that the decision of informal payments may be made by household rather than individual. Third, we used hierarchical propensity score matching analysis to assess potential selection bias. Finally, we tested the sampling age deviation to assess potential bias that our estimates were driven by particular age population. Due to space limit, results of the first and the third robustness checks are reported, and those of the other tests can be found in the appendix Tables 9 and 10.

The estimates may still have been biased by observable factors correlated with health insurance and with subsequent informal payment decisions. N Nunn and L Wantchekon [54] elucidated that selection on observables can be used to assess potential bias from unobservables. A standard protocol has been developed to gauge the strength of the possible bias arising from unobservables by calculating the coefficient of restricted regression (${\hat{\beta}}^R$) and the coefficient of unrestricted estimation (${\hat{\beta}}^F$). The results are presented in the form of ${\hat{\beta}}^F/\left({\hat{\beta}}^R-{\hat{\beta}}^F\right)$, with smaller values indicating a lower chance of bias contaminated by unobserved factors.

We considered two sets of restricted estimates: one with no controls except for the provincial fixed effect, and another with a sparse set of individual controls that included age and gender only. Six combinations of the ratio ${\hat{\beta}}^F/\left({\hat{\beta}}^R-{\hat{\beta}}^F\right)$ are reported in Table 5. With the absolute value of the ratios ranging from 0.02 to 2.82, the effects of missing variables are controllable according to N Nunn and L Wantchekon [54]. Therefore, our statistical results presented above are robust.

**Table 5:** Using selection on observables to assess the bias from unobservables

	SHI		PHI		Ins	
	None	Gender + Age	None	Gender + Age	None	Gender + Age
Red envelopes	−1.07	−1.16	−1.53	0.72	−0.87	−1.03

The hierarchical propensity score matching method suggested by A Wagstaff, M Lindelow, J Gao, L Xu and J Qian [36] and Y Chen and GZ Jin [39] was also performed to corroborate the associations identified with regard to potential selection bias. We divided the full sample into three groups: Group I covered by SHI only, Group II covered by both SHI and PHI, and the uninsured Group III. The propensity of Group I was compared with that of Group II and Group III in order to correct the endogenous take-up of health insurance due to unobservables [39]. Table 6 reports the match estimates, and Table 7 presents the treatment effect after matching. Both sets of results are consistent with our findings reported in Table 2 and Table 3 and hence corroborate our estimation.

**Table 6:** Results of the propensity score matching

	SHI = 1		PHI = 1		SHI = 1 & PHI = 1		SHI = 1 & PHI = 0	
	ATT	T-stat	ATT	T-stat	ATT	T-stat	ATT	T-stat
Red envelopes	0.02	1.43	0.00	0.00	0.00	0.00	0.01	0.84

**Table 7:** Estimates of variation in treatment effects

	Red envelopes
Panel A: *treat*_*1*_ *= SHI*	
Treat_1_ × 2013	3.549^***^
	(0.475)
treat_1_ × 2015	3.863^***^
	(0.505)
N	1642
Panel B: *treat*_*2*_ *= SHI×PHI*	
treat_*2*_ × 2013	−0.056
	(0.570)
treat_*2*_ × 2015	0.022
	(0.580)
N	1555
Province	Yes
Time	Yes
Prov×Time	Yes
Control	Yes

Note: * = *p* < 0.1, ** = *p* < 0.05, *** = *p* < 0.01; robust standard errors in parentheses

## Discussion

This article provides empirical evidence for the scholarly debate on whether health insurance reforms are able to curb informal payments in a health system. We examine the impact of health insurance coverage on patients’ behavior of making red envelope payments, the form of informal payments in China. By analyzing a nationwide panel dataset, this study finds that health insurance status indeed has a marked effect on patients’ decision to make informal payments. More important, this effect is distinct between SHI and PHI, as well as between different SHI programs. Specifically, SHI coverage significantly increases the probability of patients making informal payments in the setting of inpatient care, while PHI status significantly reduces that probability. The probability of patients making informal payments is significantly increased when they are covered by both SHI and PHI, and this increase is statistically greater than that when the person is insured by SHI only.

We interpret the empirical findings with the concept of budget constraint. In a health system characterized by the unequal allocation of medical resources, the dual pursuit of cost saving and quality care may drive patients to make informal payments for personal gains. By relaxing the budget constraints of patients through the provision of financial protection, health insurance coverage may further encourage patients to make such payments. Informal payments become possible when the benefits, such as faster access to better care, are perceived to outweigh the monetary costs. The prevalence of these transactions is aggravated by the decline in medical ethics, the poor enforcement of the professional code of conduct, the low remuneration of physicians, and powerful informal societal rules [7, 9].

## Conclusion

In this study, we engage the emerging literature that explores the association between health insurance coverage and informal payments. The case of China provides an ideal laboratory to do so. Our findings reveal that the rapid expansion of health insurance has not been able to curb rampant informal payments in the Chinese health system; ironically, it has fueled this type of transaction. Reinforcing the long-recognized phenomenon of insurance-induced demands [55], the findings derived from this study elucidate that health insurance coverage may also induce informal payments outside the formal health system. Our study suggests that the introduction of market mechanisms such as competition and PHI programs may help reduce the payment of red envelopes.

As an informal social institution—one of the rules of the game that shape people’s behaviors—red envelopes interact with formal institutions, especially the health insurance system, in a dynamic way. Policy efforts aimed at curbing informal payments must therefore be based on a thorough understanding of their complex socioeconomic causes and attack the perverse incentives in a coherent and bona fide manner. In this sense, this study offers useful lessons for other developing countries experiencing health insurance expansion and the phenomenon of rampant informal payments.

## Data Availability

The original dataset is available from the corresponding author upon request.

## Appendix

**Table 8:** Definition of variables

Variables	Description
Dependent variables	
Red envelopes	Whether or not a red envelope payment was made in the most recent inpatient admission
Independent variables	
ins	Involved in any health insurance? Yes = 1, No = 0
SHI	Involved in any social health insurance? Yes = 1, No = 0
PHI	Involved in any commercial health insurance? Yes = 1, No = 0
UEBMI	Involved in UEMI? Yes = 1, No = 0
NCMS	Involved in NCMS? Yes = 1, No = 0
Tins	Interaction between year and SHI coverage
controls	
gender	Male = 1, female = 0
age	Age of the subjects
married	Married = 1, unmarried = 2, other = 3
hukou	Rural = 1, urban = 2, other = 3
rural	Live in rural or urban region. Rural = 1, urban = 0
health status	Health status
household size	Household size
ln (income)	Ln (individual annual income)
ln (asset)	Ln (household annual assets)
work status	Job categories
*N*	Sample size

**Table 9:** Estimates with subsamples separated by age = 60

	60+				60-			
	(1)	(2)	(3)	(4)	(5)	(6)	(7)	(8)
	Inpatient				Outpatient			
gov	0.03		−0.019	−0.034	0.013**		0.013**	0.013**
	(0.032)		(0.030)	(0.032)	(0.006)		(0.006)	(0.006)
priv		−0.164*	−0.177**	− 0.225**		0.013	0.015	0.011
		(0.085)	(0.087)	(0.114)		(0.018)	(0.018)	(0.007)
gov × priv				0.182				0.004
				(0.121)				(0.021)
N	1048	1044	1044	1044	1733	1732	1732	1732
Province	Yes	Yes	Yes	Yes	Yes	Yes	Yes	Yes
Time	Yes	Yes	Yes	Yes	Yes	Yes	Yes	Yes
Prov×Time	Yes	Yes	Yes	Yes	Yes	Yes	Yes	Yes
Control	Yes	Yes	Yes	Yes	Yes	Yes	Yes	Yes

Note: **p* < 0.1, ***p* < 0.05, ****p* < 0.01; Robust standard error in parentheses. Impact of outpatient care for group aged above 60 and of inpatient care for group age below 60 are not estimated due to limited sample size

**Table 10:** Estimates with the household data

	(1)	(2)	(3)	(4)
gov	0.002		0.002	0.001
	(0.009)		(0.009)	(0.010)
priv		0.007	0.007	−0.024
		(0.016)	(0.016)	(0.038)
gov × priv				0.039
				(0.042)
N	3639	3633	3632	3632
Province	Yes	Yes	Yes	Yes
Time	Yes	Yes	Yes	Yes
Prov×Time	Yes	Yes	Yes	Yes
Control	Yes	Yes	Yes	Yes

Note: * = *p* < 0.1, ** = *p* < 0.05, *** = *p* < 0.01; Robust standard error in parentheses

## Abbreviations

ADL: Activities of Daily Living
CHARLS: The China Health and Retirement Longitudinal Survey
CMS: The Cooperative Medical Scheme
GIS: The Government Insurance Scheme
LIS: The Labor Insurance Scheme
LPM: Linear probability model
NCMS: The New Cooperative Medical Scheme
PHI: Private Health Insurance
SHI: Social health insurance
UEBMI: The Urban Employee Basic Medical Insurance Scheme
URBMI: The Urban Resident Basic Medical Insurance Scheme
URRBMI: The Urban-Rural Resident Basic Medical Insurance Scheme

## References

[1] Lewis M (2007). Informal payments and the financing of health care in developing and transition countries. Health Aff (Millwood).

[2] Baji P, Rubashkin N, Szebik I, Stoll K, Vedam S (2017). Informal cash payments for birth in Hungary: are women paying to secure a known provider, respect, or quality of care?. Soc Sci Med.

[3] Chereches RM, Ungureanu MI, Sandu P, Rus IA (2013). Defining informal payments in healthcare: a systematic review. Health Policy.

[4] Gaal P, McKee M (2004). Informal payment for health care and the theory of ‘INXIT’. Int J Health Plann Manag.

[5] Souliotis K, Golna C, Tountas Y, Siskou O, Kaitelidou D, Liaropoulos L (2016). Informal payments in the Greek health sector amid the financial crisis: old habits die last. Eur J Health Econ.

[6] Yang J (2013). The impact of informal payments on quality and equality in the Chinese health care system: a study from the perspective of doctors. Health Sociol Rev.

[7] Cohen N (2012). Informal payments for health care – the phenomenon and its context. Health Econ Policy Law.

[8] Williams CC, Horodnic AV (2017). Rethinking informal payments by patients in Europe: an institutional approach. Health Policy.

[9] Lindkvist I (2013). Informal payments and health worker effort: a quantitative study from Tanzania. Health Econ.

[10] Zhou H, Zhang J (2004). Analysis of the “red packet” phenomenon in China’s medical health industry. China & World Economy.

[11] Zhu W, Wang L, Yang C (2018). Corruption or professional dignity: an ethical examination of the phenomenon of “red envelopes” (monetary gifts) in medical practice in China. Dev World Bioeth.

[12] Kankeu HT, Ventelou B (2016). Socioeconomic inequalities in informal payments for health care: an assessment of the ‘Robin Hood’ hypothesis in 33 African countries. Soc Sci Med.

[13] Tambor M, Pavlova M, Golinowska S, Sowada C, Groot W (2013). The formal–informal patient payment mix in European countries. Governance, economics, culture or all of these?. Health Policy.

[14] Atanasova E, Pavlova M, Moutafova E, Rechel B, Groot W (2014). Informal payments for health services: the experience of Bulgaria after 10 years of formal co-payments. Eur J Pub Health.

[15] Chiu Y-C, Smith KC, Morlock L, Wissow L (2007). Gifts, bribes and solicitions: print media and the social construction of informal payments to doctors in Taiwan. Soc Sci Med.

[16] Meng Q, Xu L, Zhang Y, Qian J, Cai M, Xin Y, Gao J, Xu K, Boerma JT, Barber SL (2012). Trends in access to health services and financial protection in China between 2003 and 2011: a cross-sectional study. Lancet.

[17] Meng Q, Fang H, Liu X, Yuan B, Xu J (2015). Consolidating the social health insurance schemes in China: towards an equitable and efficient health system. Lancet.

[18] He AJ, Wu S (2017). Towards universal health coverage via social health Insurance in China: systemic fragmentation, reform imperatives, and policy alternatives. App Health Econ Health Policy.

[19] Li X, Lu J, Hu S, Cheng KK, De Maeseneer J, Meng Q, Mossialos E, Xu DR, Yip W, Zhang H (2017). The primary health-care system in China. Lancet.

[20] Hsiao WCL, Liu Y (1996). Economic reform and health — lessons from China. N Engl J Med.

[21] Zhou H, Zhang J (2004). A social historical analysis of "red envelope" in medical services in China. Chinese J Population Sci.

[22] Eggleston K, Ling L, Qingyue M, Lindelow M, Wagstaff A (2008). Health service delivery in China: a literature review. Health Econ.

[23] Liu Y (2002). Reforming China's urban health insurance system. Health Policy.

[24] Meng Q, Tang S (2013). Universal health care coverage in China: challenges and opportunities. Procedia - Social Behavioral Sci.

[25] Hsiao WCL (1995). The Chinese health care system: lessons for other nations. Soc Sci Med.

[26] Yip W, Hsiao W (2009). China's health care reform: a tentative assessment. China Econ Rev.

[27] Kong X, Du Z, Zhao M (2011). Red envelopes and doctor-patient trust: report of research on national questionnaire survey of 4000 inpatients in 10 cities (VII). Medicine Philosophy.

[28] Guo Y (2015). Personal relationships with the physicians, impression on the physicians and the urban residents' residents’ behavior of sending the red envelopes. Chinese Health Service Management.

[29] Liu GG, Vortherms AS, Hong X (2017). China's health reform update. Annu Rev Public Health.

[30] Yip W, Hsiao W, Meng Q, Chen W, Sun X (2010). Realignment of incentives for health-care providers in China. Lancet.

[31] He AJ (2014). The doctor-patient relationship, defensive medicine and overprescription in Chinese public hospitals: evidence from a cross-sectional survey in Shenzhen city. Soc Sci Med.

[32] Cabral M, Geruso M, Mahoney N (2018). Do larger health insurance subsidies benefit patients or producers? Evidence from Medicare advantage. Am Econ Rev.

[33] Finkelstein A, Taubman S, Wright B, Bernstein M, Gruber J, Newhouse JP, Allen H, Baicker K (2012). The Oregon health insurance experiment: evidence from the first year. Quart J Econ.

[34] Koijen RSJ, Van Nieuwerburgh S, Yogo M (2016). Health and Mortality Delta: assessing the welfare cost of household insurance choice. J Financ.

[35] Cutler DM, Zeckhauser RJ, Culyer A, Newhouse J (2000). The Anatomy of Health Insurance. Handbook of Health Economics.

[36] Wagstaff A, Lindelow M, Gao J, Xu L, Qian J (2009). Extending health insurance to the rural population: an impact evaluation of China's new cooperative medical scheme. J Health Econ.

[37] Hubbard RG, Skinner J, Zeldes SP (1995). Precautionary saving and social insurance. J Polit Economy.

[38] Currie J, Gruber J (1996). Health insurance eligibility, utilization of medical care, and child health. Q J Econ.

[39] Chen Y, Jin GZ (2012). Does health insurance coverage lead to better health and educational outcomes? Evidence from rural China. J Health Econ.

[40] Ward L, Franks P (2007). Changes in health care expenditure associated with gaining or losing health insurance. Ann Intern Med.

[41] Liu H, Gao S, Rizzo JA (2011). The expansion of public health insurance and the demand for private health insurance in rural China. China Econ Rev.

[42] Rickne J (2013). Labor market conditions and social insurance in China. China Econ Rev.

[43] Chen L, Wang J, Fan H (2018). Health insurance and social integration of migrant population: evidence from commercial insurance. Insurance Studies.

[44] Jung J, Streeter LJ (2015). Does health insurance decrease health expenditure risk in developing countries? The case of China. Southern Econ J.

[45] Zhang A, Nikoloski Z, Mossialos E (2017). Does health insurance reduce out-of-pocket expenditure? Heterogeneity among China's middle-aged and elderly. Soc Sci Med.

[46] Liaropoulos L, Siskou O, Kaitelidou D, Theodorou M, Katostaras T (2008). Informal payments in public hospitals in Greece. Health Policy.

[47] Xie Y, Gu X (2018). Adverse selection and its influencing factors of middle- and old-aged people in China’s social health insurance. Insurance Studies.

[48] Peng H, Zheng Q, Guo Y (2017). Does the expansion of social health insurance in China promote the development of private health insurance?. J Finan Res.

[49] Björnberg A, Phang YA (2018). Euro health consumer index 2018.

[50] Zhao Y, Hu Y, Strauss J, Smith JP, Yang G (2012). Cohort profile: the China health and retirement longitudinal study (CHARLS). Int J Epidemiol.

[51] Feng X, Pang M, Beard J (2014). Health system strengthening and hypertension awareness, treatment and control: data from the China health and retirement longitudinal study. Bull World Health Organ.

[52] Wang S, Chen R, Liu Q, Zhan S, Li L (2015). Comprehensive treatment of hypertension middle-aged and elderly people: cross-sectional survey data from the China health and retirement longitudinal study (CHARLS). Lancet.

[53] Lei X, Sun X, Strauss J, Zhang P, Zhao Y (2014). Depressive symptoms and SES among the mid-aged and elderly in China: evidence from the China health and retirement longitudinal study national baseline. Soc Sci Med.

[54] Nunn N, Wantchekon L (2011). The slave trade and the origins of mistrust in Africa. Am Econ Rev.

[55] McGuire TG, Culyer AJ, Newhouse JP (2000). Physician agency. In: handbook of health economics.

